# Supplementary material for: Development of a computer-aided design software for the quantitative evaluation of aesthetic damage
Source: PLoS One. 2019 Dec 18;14(12):e0226322. doi: 10.1371/journal.pone.0226322 (PMC6919621; doi:10.1371/journal.pone.0226322)
Supplement: S1 Code site — 1. (ZIP) [file pone.0226322.s001.zip › [EESC_jr][Projeto]Relatorio_07_21/reports/templates_zip/gentelella-master.zip!/gentelella-master/vendors/DateJS/reports/lcov-report/core/format_parser.js.html]

Code coverage report for core/format\_parser.js


# Code coverage report for core/format\_parser.js

## Statements: 96.95% (159 / 164)      Branches: 86.13% (118 / 137)      Functions: 94.29% (33 / 35)      Lines: 96.95% (159 / 164)      Ignored: none

All files » core/ » format\_parser.js

```
|  |  |  |
| --- | --- | --- |
| 1 2 3 4 5 6 7 8 9 10 11 12 13 14 15 16 17 18 19 20 21 22 23 24 25 26 27 28 29 30 31 32 33 34 35 36 37 38 39 40 41 42 43 44 45 46 47 48 49 50 51 52 53 54 55 56 57 58 59 60 61 62 63 64 65 66 67 68 69 70 71 72 73 74 75 76 77 78 79 80 81 82 83 84 85 86 87 88 89 90 91 92 93 94 95 96 97 98 99 100 101 102 103 104 105 106 107 108 109 110 111 112 113 114 115 116 117 118 119 120 121 122 123 124 125 126 127 128 129 130 131 132 133 134 135 136 137 138 139 140 141 142 143 144 145 146 147 148 149 150 151 152 153 154 155 156 157 158 159 160 161 162 163 164 165 166 167 168 169 170 171 172 173 174 175 176 177 178 179 180 181 182 183 184 185 186 187 188 189 190 191 192 193 194 195 196 197 198 199 200 201 202 203 204 205 206 207 208 209 210 211 212 213 214 215 216 217 218 219 220 221 222 223 224 225 226 227 228 229 230 231 232 233 234 235 236 237 238 239 240 241 242 243 244 245 246 247 248 249 250 251 252 253 254 255 256 257 258 259 260 261 262 263 264 265 266 267 268 269 270 271 272 273 274 275 276 277 278 279 280 281 282 283 284 285 286 287 288 289 290 291 292 293 294 295 296 297 298 299 300 301 302 303 304 305 306 307 308 309 310 311 312 313 314 315 316 317 318 319 320 321 322 323 324 325 326 327 328 329 330 331 332 333 334 335 336 337 338 339 340 341 342 343 344 345 346 347 348 349 350 351 352 353 354 355 356 357 358 359 360 361 362 363 364 365 366 367 368 369 370 371 372 373 374 375 376 377 378 379 380 381 | 1   1   39302     1 1         1 84     1   530 530 13780 13780 13780     13780   13780     530     17 17 17 17 17 17 17     31 17   31 403 31 31   372     31     21 21   16   5 5 2   5   21 21     84 84 84 84 84 84   53 53   84     960 960 400 240   160   560 400   160       80 80 1200 1200     80 8 8 4 4 4   8   80     80                                       48     17 17     52     40     29                                   80     160 160 160 160 1440   160     1120     1440 320 1120 1120         160 160 160 1440 1120   320     160       1 84   84 84   84 31   53     84   84 21   84     1     454 454 374     80   80     80       1 573     375     375   368   7 1 1   6 6 2   4 12   4 4   4 4   4 4     4       1   160 160                         160 160                                           160                         160 160 160 160   160   5       4 4               160 160     530 2120   530     530 530   530 530 530                       530     1 | ``` (function () { 	"use strict"; 	Date.Parsing = { 		Exception: function (s) { 			this.message = "Parse error at '" + s.substring(0, 10) + " ...'"; 		} 	}; 	var $P = Date.Parsing; 	var dayOffsets = { 		standard: [0,31,59,90,120,151,181,212,243,273,304,334], 		leap: [0,31,60,91,121,152,182,213,244,274,305,335] 	};   	$P.isLeapYear = function(year) { 		return ((year % 4 === 0) && (year % 100 !== 0)) \|\| (year % 400 === 0); 	};   	var utils = { 		multiReplace : function (str, hash ) { 			var key; 			for (key in hash) { 				Eif (Object.prototype.hasOwnProperty.call(hash, key)) { 					var regex; 					Iif (typeof hash[key] === "function") {   					} else { 						regex = (hash[key] instanceof RegExp) ? hash[key] : new RegExp(hash[key], "g"); 					} 					str = str.replace(regex, key); 				} 			} 			return str; 		}, 		getDayOfYearFromWeek : function (obj) { 			var d, jan4, offset; 			obj.weekDay = (!obj.weekDay && obj.weekDay !== 0) ? 1 : obj.weekDay; 			d = new Date(obj.year, 0, 4); 			jan4 = d.getDay() === 0 ? 7 : d.getDay(); // JS is 0 indexed on Sunday. 			offset = jan4+3; 			obj.dayOfYear = ((obj.week * 7) + (obj.weekDay === 0 ? 7 : obj.weekDay))-offset; 			return obj; 		}, 		getDayOfYear : function (obj, dayOffset) { 			if (!obj.dayOfYear) { 				obj = utils.getDayOfYearFromWeek(obj); 			} 			for (var i=0;i <= dayOffset.length;i++) { 				if (obj.dayOfYear < dayOffset[i] \|\| i === dayOffset.length) { 					obj.day = obj.day ? obj.day : (obj.dayOfYear - dayOffset[i-1]); 					break; 				} else { 					obj.month = i; 				} 			} 			return obj; 		}, 		adjustForTimeZone : function (obj, date) { 			var offset; 			if (obj.zone.toUpperCase() === "Z" \|\| (obj.zone_hours === 0 && obj.zone_minutes === 0)) { 				// it's UTC/GML so work out the current timeszone offset 				offset = -date.getTimezoneOffset(); 			} else { 				offset = (obj.zone_hours*60) + (obj.zone_minutes \|\| 0); 				if (obj.zone_sign === "+") { 					offset *= -1; 				} 				offset -= date.getTimezoneOffset(); 			} 			date.setMinutes(date.getMinutes()+offset); 			return date; 		}, 		setDefaults : function (obj) { 			obj.year = obj.year \|\| Date.today().getFullYear(); 			obj.hours = obj.hours \|\| 0; 			obj.minutes = obj.minutes \|\| 0; 			obj.seconds = obj.seconds \|\| 0; 			obj.milliseconds = obj.milliseconds \|\| 0; 			if (!(!obj.month && (obj.week \|\| obj.dayOfYear))) { 				// if we have a month, or if we don't but don't have the day calculation data 				obj.month = obj.month \|\| 0; 				obj.day = obj.day \|\| 1; 			} 			return obj; 		}, 		dataNum: function (data, mod, explict, postProcess) { 			var dataNum = data*1; 			if (mod) { 				if (postProcess) { 					return data ? mod(data)*1 : data; 				} else { 					return data ? mod(dataNum) : data; 				} 			} else if (!explict){ 				return data ? dataNum : data; 			} else { 				return (data && typeof data !== "undefined") ? dataNum : data; 			} 		}, 		timeDataProcess: function (obj) { 			var timeObj = {}; 			for (var x in obj.data) { 				Eif (obj.data.hasOwnProperty(x)) { 					timeObj[x] = obj.ignore[x] ? obj.data[x] : utils.dataNum(obj.data[x], obj.mods[x], obj.explict[x], obj.postProcess[x]); 				} 			} 			if (obj.data.secmins) { 				obj.data.secmins = obj.data.secmins.replace(",", ".") * 60; 				if (!timeObj.minutes) { 					timeObj.minutes = obj.data.secmins; 				} else Eif (!timeObj.seconds) { 					timeObj.seconds = obj.data.secmins; 				} 				delete obj.secmins; 			} 			return timeObj; 		}, 		buildTimeObjectFromData: function (data) { 			var time = utils.timeDataProcess({ 				data: { 					year : data[1], 					month : data[5], 					day : data[7], 					week : data[8], 					dayOfYear : data[10], 					hours : data[15], 					zone_hours : data[23], 					zone_minutes : data[24], 					zone : data[21], 					zone_sign : data[22], 					weekDay : data[9], 					minutes: data[16], 					seconds: data[19], 					milliseconds: data[20], 					secmins: data[18] 				}, 				mods: { 					month: function(data) { 						return data-1; 					}, 					weekDay: function (data) { 						data = Math.abs(data); 						return (data === 7 ? 0 : data); 					}, 					minutes: function (data) { 						return data.replace(":",""); 					}, 					seconds: function (data) { 						return Math.floor( (data.replace(":","").replace(",","."))*1 ); 					}, 					milliseconds: function (data) { 						return (data.replace(",",".")*1000); 					} 				}, 				postProcess: { 					minutes: true, 					seconds: true, 					milliseconds: true 				}, 				explict: { 					zone_hours: true, 					zone_minutes: true 				}, 				ignore: { 					zone: true, 					zone_sign: true, 					secmins: true 				} 			}); 			return time; 		}, 		addToHash: function (hash, keys, data) { 			keys = keys; 			data = data; 			var len = keys.length; 			for (var i = 0; i < len; i++) { 			  hash[keys[i]] = data[i]; 			} 			return hash; 		}, 		combineRegex: function (r1, r2) { 			return new RegExp("(("+r1.source+")\\s("+r2.source+"))"); 		}, 		getDateNthString: function(add, last, inc){ 			if (add) { 				return Date.today().addDays(inc).toString("d"); 			} else Eif (last) { 				return Date.today().last()[inc]().toString("d"); 			} 			 		}, 		buildRegexData: function (array) { 			var arr = []; 			var len = array.length; 			for (var i=0; i < len; i++) { 				if (Array.isArray(array[i])) { 					arr.push(this.combineRegex(array[i][0], array[i][1])); 				} else { 					arr.push(array[i]); 				} 			} 			return arr; 		} 	};   	$P.processTimeObject = function (obj) { 		var date, dayOffset;   		utils.setDefaults(obj); 		dayOffset = ($P.isLeapYear(obj.year)) ? dayOffsets.leap : dayOffsets.standard;   		if (!obj.month && (obj.week \|\| obj.dayOfYear)) { 			utils.getDayOfYear(obj, dayOffset); 		} else { 			obj.dayOfYear = dayOffset[obj.month] + obj.day; 		}   		date = new Date(obj.year, obj.month, obj.day, obj.hours, obj.minutes, obj.seconds, obj.milliseconds);   		if (obj.zone) { 			utils.adjustForTimeZone(obj, date); // adjust (and calculate) for timezone 		} 		return date; 	}; 	 	$P.ISO = { 		regex : /^([\+-]?\d{4}(?!\d{2}\b))((-?)((0[1-9]\|1[0-2])(\3([12]\d\|0[1-9]\|3[01]))?\|W([0-4]\d\|5[0-3])(-?[1-7])?\|(00[1-9]\|0[1-9]\d\|[12]\d{2}\|3([0-5]\d\|6[1-6])))([T\s]((([01]\d\|2[0-4])((:?)[0-5]\d)?\|24\:?00)([\.,]\d+(?!:))?)?(\17[0-5]\d([\.,]\d+)?)?\s?([zZ]\|([\+-])([01]\d\|2[0-3]):?([0-5]\d)?)?)?)?$/, 		parse : function (s) { 			var time, data = s.match(this.regex); 			if (!data \|\| !data.length) { 				return null; 			}   			time = utils.buildTimeObjectFromData(data);   			Iif (!time.year \|\| (!time.year && (!time.month && !time.day) && (!time.week && !time.dayOfYear)) ) { 				return null; 			} 			return $P.processTimeObject(time); 		} 	};   	$P.Numeric = { 		isNumeric: function (e){return!isNaN(parseFloat(e))&&isFinite(e);}, 		regex: /\b([0-1]?[0-9])([0-3]?[0-9])([0-2]?[0-9]?[0-9][0-9])\b/i, 		parse: function (s) { 			var data, i, 				time = {}, 				order = Date.CultureInfo.dateElementOrder.split(""); 			if (!(this.isNumeric(s)) \|\| // if it's non-numeric OR 				(s[0] === "+" && s[0] === "-")) {			// It's an arithmatic string (eg +/-1000) 				return null; 			} 			if (s.length < 5 && s.indexOf(".") < 0 && s.indexOf("/") < 0) { // assume it's just a year. 				time.year = s; 				return $P.processTimeObject(time); 			} 			data = s.match(this.regex); 			if (!data \|\| !data.length) { 				return null; 			} 			for (i=0; i < order.length; i++) { 				switch(order[i]) { 					case "d": 						time.day = data[i+1]; 						break; 					case "m": 						time.month = (data[i+1]-1); 						break; 					case "y": 						time.year = data[i+1]; 						break; 				} 			} 			return $P.processTimeObject(time); 		} 	};   	$P.Normalizer = { 		regexData: function () { 			var $R = Date.CultureInfo.regexPatterns; 			return utils.buildRegexData([ 				$R.tomorrow, 				$R.yesterday, 				[$R.past, $R.mon], 				[$R.past, $R.tue], 				[$R.past, $R.wed], 				[$R.past, $R.thu], 				[$R.past, $R.fri], 				[$R.past, $R.sat], 				[$R.past, $R.sun] 			]); 		}, 		basicReplaceHash : function() { 			var $R = Date.CultureInfo.regexPatterns; 			return { 				"January": $R.jan.source, 				"February": $R.feb, 				"March": $R.mar, 				"April": $R.apr, 				"May": $R.may, 				"June": $R.jun, 				"July": $R.jul, 				"August": $R.aug, 				"September": $R.sep, 				"October": $R.oct, 				"November": $R.nov, 				"December": $R.dec, 				"": /\bat\b/gi, 				" ": /\s{2,}/, 				"am": $R.inTheMorning, 				"9am": $R.thisMorning, 				"pm": $R.inTheEvening, 				"7pm":$R.thisEvening 			}; 		}, 		keys : function(){ 			return [ 				utils.getDateNthString(true, false, 1),				// tomorrow 				utils.getDateNthString(true, false, -1),			// yesterday 				utils.getDateNthString(false, true, "monday"),		//last mon 				utils.getDateNthString(false, true, "tuesday"),		//last tues 				utils.getDateNthString(false, true, "wednesday"),	//last wed 				utils.getDateNthString(false, true, "thursday"),	//last thurs 				utils.getDateNthString(false, true, "friday"),		//last fri 				utils.getDateNthString(false, true, "saturday"),	//last sat 				utils.getDateNthString(false, true, "sunday")		//last sun 			]; 		}, 		buildRegexFunctions: function () { 			var $R = Date.CultureInfo.regexPatterns; 			var __ = Date.i18n.__; 			var tomorrowRE = new RegExp("(\\b\\d\\d?("+__("AM")+"\|"+__("PM")+")? )("+$R.tomorrow.source.slice(1)+")", "i"); // adapted tomorrow regex for AM PM relative dates 			var todayRE = new RegExp($R.today.source + "(?!\\s*([+-]))\\b"); // today, but excludes the math operators (eg "today + 2h") 			 			this.replaceFuncs = [ 				[todayRE, function (full) { 					return (full.length > 1) ? Date.today().toString("d") : full; 				}], 				[tomorrowRE, 				function(full, m1) { 					var t = Date.today().addDays(1).toString("d"); 					return (t + " " + m1); 				}], 				[$R.amThisMorning, function(str, am){return am;}], 				[$R.pmThisEvening, function(str, pm){return pm;}] 			]; 				 		}, 		buildReplaceData: function () { 			this.buildRegexFunctions(); 			this.replaceHash = utils.addToHash(this.basicReplaceHash(), this.keys(), this.regexData()); 		}, 		stringReplaceFuncs: function (s) { 			for (var i=0; i < this.replaceFuncs.length; i++) { 				s = s.replace(this.replaceFuncs[i][0], this.replaceFuncs[i][1]); 			} 			return s; 		}, 		parse: function (s) { 			s = this.stringReplaceFuncs(s); 			s = utils.multiReplace(s, this.replaceHash);   			try { 				var n = s.split(/([\s\-\.\,\/\x27]+)/); 				Iif (n.length === 3 && 					$P.Numeric.isNumeric(n[0]) && 					$P.Numeric.isNumeric(n[2]) && 					(n[2].length >= 4)) { 						// ok, so we're dealing with x/year. But that's not a full date. 						// This fixes wonky dateElementOrder parsing when set to dmy order. 						if (Date.CultureInfo.dateElementOrder[0] === "d") { 							s = "1/" + n[0] + "/" + n[2]; // set to 1st of month and normalize the seperator 						} 				} 			} catch (e) {}   			return s; 		} 	}; 	$P.Normalizer.buildReplaceData(); }()); ``` |
```

Generated by istanbul at Sun Nov 16 2014 12:08:33 GMT-0800 (PST)
